# Acknowledgement of manuscript reviewers 2014

**DOI:** 10.1186/s40200-015-0128-3

**Published:** 2015-02-25

**Authors:** Bagher Larijani, Farnoush Faridbod, Seyed Masoud Arzaghi, Fatemeh Bandarian, Azadeh Ebrahim-Habibi, Hossein Fakhrzadeh, Shirin Hasani-Ranjbar, Ramin Heshmat, Abbas Ali Keshtkar, Patricia Khashayar, Ensieh Nasli-Esfahani, Farshad Sharifi, Akbar Soltani, Farzaneh Zahedi

**Affiliations:** Tehran University of Medical Sciences, Tehran, Iran

## Abstract

The editors of *Journal of Diabetes & Metabolic Disorders* would like to thank all our reviewers who have contributed to the journal in Volume 13 (2014).

**Ebrahim Abbasi**

Iran

**Farzaneh Abbasi**

Iran

**Aly Abdel Rahim**

Egypt

**Majedah Abdul-Rasoul**

Kuwait

**Mohamadsadegh Abedzadeh Zavareh**

Iraq

**Wichai Aekplakorn**

Thailand

**Lucie Agosta**

United States of America

**Tarunveer Ahluwalia**

Denmark

**Aliasghar Ahmad Kiadaliri**

Sweden

**Muhammad Yakoob Ahmedani**

United States of America

**James Aikens**

United States of America

**Hagop Akiskal**

United States of America

**Onur Akpinar**

Turkey

**Hassan Akrami**

Iran

**Sudabeh Alatab**

Iran

**Mahtab Alizadeh-Khoei**

Iran

**Edurne Alonso Morán**

Spain

**Dr. Abdulbasit Al-Sieni**

Saudi Arabia

**Pedro Andrade**

Portugal

**Zohreh Annabestani**

Iran

**Slobodan Apostolski**

Serbia

**Maryam Ardeshiri**

Iran

**Tahereh Arefirad**

Iran

**Babak Arjmand**

Iran

**Carolina Arruabarrena**

Spain

**Benoit Arsenault**

Netherlands

**Hamid Asayesh**

Iran

**Ambika Ashraf**

United States of America

**Stephen Atkin**

Qatar

**Georg Auburger**

Germany

**Ata Allah Bagherzadeh**

Iran

**Zahra Bahadoran**

Iran

**Farid Bahrpeyma**

Iran

**Harpreet Bajaj**

Canada

**Fatemeh Bandarian**

Iran

**Nidhi Bansal**

United States of America

**Hamid Reza Baradaran**

Iran

**Manash P Baruah**

India

**Ali Barzegari**

Iran

**Joshua Barzilay**

United States of America

**Randall Basaraba**

United States of America

**Murat Bastepe**

United States of America

**Lourdes Basurto**

Mexico

**Nurcan Bektas**

Turkey

**Kirstine Bell**

Australia

**Samir Ben Ali**

Tunisia

**Antonino Bianco**

Italy

**Robin Callister**

Australia

**Ana Elisa Castro-Sánchez**

Mexico

**Alper Celik**

Turkey

**Mustafa Cetin**

Turkey

**Tien-Chun Chang**

Taiwan

**Roberta Cobas**

Brazil

**Paul de vos**

Netherlands

**Michelle DeGeeter**

United States of America

**Maria Delbin**

Brazil

**Muralikrishnan Dhanasekaran**

United States of America

**Shirin Djalalinia**

Iran

**Yavuz Dodurga**

Turkey

**Azadeh Ebrahim-Habibi**

Iran

**Mehdi Ebrahimi**

Iran

**Mehdi Ebrahimi**

Iraq

**Mohammad Hassan Eftekhari**

Iran

**Elhassan Elhassan**

Sudan

**Rajiv Erasmus**

South Africa

**Hossein Fakhrzadeh**

Iran

**Mohammad-Sadegh Fallah**

Iran

**Farshad Farzadfar**

Iran

**Vassilis Filiopoulos**

Greece

**Mahboubeh Firouzkouhi Moghaddam**

Iran

**Bijan Forogh**

Iran

**Maria Pia Francescato**

Italy

**Xiaobing Fu**

China

**Jeffrey Gergley**

United States of America

**Magdalena Gershater**

Sweden

**Asghar Ghasemi**

Iran

**Saeid Ghavami**

Canada

**Maryam Ghodsi**

Iran

**Marcin Gierach**

Poland

**Mukesh Gohel**

India

**Elisabeth Govers**

Netherlands

**Domenico Greco**

Italy

**Vanessa Ha**

Canada

**Monir Sadat Haerian**

Iran

**Vahid Haghpanah**

Iran

**Sakineh Hajebrahimi**

Iran

**Homa Hajimehdipoor**

Iran

**Rubina Hakeem**

Pakistan

**Weidong Han**

China

**Shahzad Syed Hasan**

Australia

**Shirin Hasani-Ranjbar**

Iran

**Mohammad Hashemi**

Iran

**Seyed-Jafar Hashemian**

Iran

**Tahereh Hassannia**

Iran

**Azita Hekmatdoost**

Iran

**Burkhard Lorenz Herrmann**

Germany

**Marisa Hilliard**

United States of America

**Jun Hosoe**

Japan

**Mostafa Hosseini**

Iran

**Arash Hossein-Nezhad**

Iran

**Kuo-Chin Huang**

Taiwan

**Zaid Ibraheem**

Iraq

**Hiroyuki Imamura**

Japan

**Urszula Iwaniec**

United States of America

**Farah Jabeen**

Pakistan

**Sehwan Jang**

Puerto Rico

**Anna Jankowska**

Poland

**Mehdi Javanbakht**

Iran

**Christie Jeon**

United States of America

**Lin Jia**

United States of America

**Takashi Joh**

Japan

**Ali Kabir**

Iran

**Dieudonne Kaimbo Wa Kaimbo**

Congo, Democratic Republic

**Rigas Kalaitzidis**

Greece

**Sanjay Kalra**

India

**Pramila Kalra**

India

**Seyyed Hamid Kamaki**

Iran

**Aditya Kapoor**

India

**Zohreh Karimi**

Iran

**Amir Kasaian**

Iran

**Zahra Kashi**

Iran

**Hideki Katoh**

Japan

**Joseph Katz**

United States of America

**Roya Kelishadi**

Iran

**Abdul Khan**

Pakistan

**Patricia Khashayar**

Iran

**Zhamak Khorgami**

United States of America

**Madhu Khullar**

India

**Davis Kibirige**

Uganda

**Piotr Kocelak**

Poland

**Fariba Kolahdooz**

Canada

**Irina Kologrivova**

Russian Federation

**Fariba Koohdani**

Iran

**Hongyu Kuang**

China

**Ajay Kumari**

India

**Gokulakrishnan Kuppan**

India

**Joan C Laguna**

Spain

**Neda Laiteerapong**

United States of America

**Ruediger Landgraf**

Germany

**Joseph Larkin**

United States of America

**Nathalie Leite**

Brazil

**Paul Loprinzi**

United States of America

**Yongjie Ma**

United States of America

**Michel Macknes**

United Kingdom

**Mohsen Maddah**

Iran

**Zhila Maghbooli**

Iran

**Mohammad Yoosef Mahjouri**

Iran

**Shideh Majidi**

United States of America

**Rayaz Malik**

United Kingdom

**Asieh Mansour**

Iran

**Xiao-Ming Mao**

China

**Luis Masana**

Spain

**Farzad Masoudkabir**

Iran

**Yorgi Mavros**

Australia

**Tina Mazaheri**

Iran

**Mairtin McDermott**

Australia

**Sarah McDonald**

Australia

**Hassan Mehrad-Majd**

Iran

**Neda Mehrdad**

Iran

**Reza Meshkani**

Iran

**Parvin Mirmiran**

Iran

**Amin Mirzaei**

Iran

**Iraj Mirzaii-Dizgah**

Iran

**Vahid Moazed**

Iran

**Kirtikumar Modi**

India

**Victor Mogre**

Ghana

**Mohammad Reza Mohajeri Tehrani**

Iran

**Masumeh Mohammadi**

Iran

**Marziyeh Mojbafan**

Iran

**Sara Mortaz Hejri**

Iran

**Shan Mou**

China

**Seyed Hadi Mousavi**

Iran

**Stephen Mshana**

Tanzania

**Soma Mukhopadhyay**

India

**Anita Munde**

India

**Shohreh Naderimagham**

Iran

**Mahdi Najafi**

Iran

**Mohsen Naseri**

Iran

**Ayat Nasrolahi**

Iran

**Neda Nayebi**

Iran

**Asfandyar Niazi**

Pakistan

**Markus Nielen**

Netherlands

**Midori Nishiyama**

Japan

**Ahmed Nooh**

Egypt

**Mitra Nourbakhsh**

Iran

**Tsuyoshi Nozue**

Japan

**Roberto Nuño-Solinís**

Spain

**Christian Okafor**

Nigeria

**Fatma Omar**

Tanzania

**Kobra Omidfar**

Iran

**Amir Pakpour**

Iran

**José Z. Parra Carrillo**

Mexico

**Siavash Parvardeh**

Iran

**Ehsan Parvaresh**

Iran

**Parvin Pasalar**

Iran

**Moloud Payab**

Iran

**Robert Peck**

Tanzania

**Maryam Peimani**

Iran

**Niloofar Peykari**

Iran

**Paolo Pinton**

Italy

**Ronald Plotnikoff**

Australia

**Mandana Pourian**

Iran

**Filipe Prazeres**

Portugal

**Purvi Purohit**

India

**Mostafa Qorbani**

Iran

**Ana R. Quinones**

United States of America

**Shadi Rahimzadeh**

Iran

**Marjan Rahnamaye Farzami**

Iran

**Krajkumar**

India

**Negin Rashidi**

Iran

**Ali Rasouli**

Iran

**Hadith Rastad**

Iran

**Farideh Razi**

Iran

**Rezvan Razmandeh**

Iran

**Philipp Rein**

Austria

**Arezoo Rezazadeh**

Iran

**Lorenz Risch**

Switzerland

**Manfredi Rizzo**

Italy

**Mehrdad Roghani**

Iran

**James Rosado**

United States of America

**Rebecca Rosenwasser**

United States of America

**Patricia Rzezak**

Brazil

**Akhmad Sabarudin**

Indonesia

**Marjan Sabbaghian**

Iran

**Christoph Saely**

Austria

**Saruta Saengtipbovorn**

Thailand

**Atoosa Saidpour**

Iran

**Pooneh Salari**

Iran

**Ana M. Salinas-Martinez**

Mexico

**Miguel Salinero-Fort**

Spain

**Ali Samadikuchaksaraei**

Iran

**Mojgan Sanjari**

Iran

**Mahnaz Sanjari**

Iran

**Fatemeh Sayarifard**

Iran

**Soroush Seifirad**

Iran

**Gunes Senol**

Turkey

**Zhaleh Shadman**

Iran

**Gita Shafiee**

Iran

**Zahra Shafieyan**

Iran

**Ali Shariati**

Iran

**Farshad Sharifi**

Iran

**Swarkar Sharma**

India

**Sara Sheikholeslami**

Iran

**Seyed Vahid Shetab-Boushehri**

Iran

**Nooshin Shirzad**

Iran

**Shahla Shojaei**

Iran

**Randhir Singh**

India

**Vinod Singh**

India

**Binayak Sinha**

India

**Wingyee So**

Hong Kong

**Ghada Soliman**

Egypt

**Mohsen Soroush**

Iran

**Rosanna Stopford**

United Kingdom

**Rona Strawbridge**

Sweden

**Krzysztof Strojek**

Poland

**Sorimuthu Subramanian**

India

**Peter Sun**

United States of America

**Tomasz Szkudelski**

Poland

**Ozra Tabatabaei-Malazy**

Iran

**Ehsaneh Taheri**

Iran

**Gholamreza Taheripak**

Iran

**Tomoka Takatani-Nakase**

Japan

**Nadine Taleb**

Canada

**Rajeev Taliyan**

India

**Akihito Tanaka**

Japan

**Solomon Tesfaye**

United Kingdom

**Vidhu Thaker**

United States of America

**Kumpei Tokuyama**

Japan

**Gerald Tomkin**

Ireland

**Tahereh Toulabi**

Iran

**Chin-Hsiao Tseng**

Taiwan

**Satoru Tsujii**

Japan

**Belma Turan**

Turkey

**Andrew Turner**

Australia

**Outi Vaarala**

Finland

**Mohammadreza Vafa**

Iran

**Mohsen Vahedi**

Iran

**Mahboubeh Vala**

Iran

**Kerri Viney**

Australia

**Fei Wang**

United States of America

**Kentaro Watanabe**

Japan

**Benjamin Wheeler**

New Zealand

**Wanghong Xu**

China

**Hemant Kumar Singh Yadav**

India

**Leila Yazdanpanah**

Iran

**Derek Yellon**

United Kingdom

**Mesfin Yimam**

United States of America

**Hoda Sadat Zahedi**

Iran

**Maryam Zahmatkesh**

Iran

**Roberto Zenteno-Cuevas**

Mexico

**Xingquan Zhao**

China

**Amir Ziaee**

Iran

**Bader Faiyaz Zuberi**

Pakistan

